# A Systematic Review of Effective Interventions and Strategies to Support the Transition of Older Adults From Driving to Driving Retirement/Cessation

**DOI:** 10.1093/geroni/igae054

**Published:** 2024-06-03

**Authors:** Anne E Dickerson, Tadhg Stapleton, Jamie Bloss, Isabelle Géinas, Priscilla Harries, Moon Choi, Isabel Margot-Cattin, Barbara Mazer, Ann-Helen Patomella, Lizette Swanepoel, Lana Van Niekerk, Carolyn A Unsworth, Brenda Vrkljan

**Affiliations:** Department of Occupational Therapy, East Carolina University, Greenville, North Carolina, USA; Discipline of Occupational Therapy, Trinity College, Dublin, Ireland; Laupus Health Sciences Library, East Carolina University, Greenville, North Carolina, USA; School of Physical and Occupational Therapy, Faculty of Medicine and Health Sciences, McGill University, Montreal, Quebec, Canada; Centre for Interdisciplinary Research in Rehabilitation of Greater Montreal, Montreal, Quebec, Canada; Graduate Research School and Researcher Development, Kingston University, London, UK; Graduate School of Science and Technology Policy, Korea Advanced Institute of Science and Technology, Daejeon, South Korea; Department of Occupational Therapy, University of Applied Sciences and Arts of Western Switzerland (HES-SO), Delémont, Switzerland; School of Physical and Occupational Therapy, Faculty of Medicine and Health Sciences, McGill University, Montreal, Quebec, Canada; Centre for Interdisciplinary Research in Rehabilitation of Greater Montreal, Montreal, Quebec, Canada; Division of Occupational Therapy, Department of Neurobiology, Karolinska Institutet, Huddinge, Sweden; Division of Occupational Therapy, Faculty of Medicine and Health Sciences, Stellenbosch University, Stellenbosch, South Africa; Division Occupational Therapy, Department of Health and Rehabilitation Sciences, Faculty of Medicine and Health Sciences, Stellenbosch University, Stellenbosch, South Africa; Institute of Health and Wellbeing, Federation University, Churchill, Victoria, Australia; School of Rehabilitation Science, Faculty of Health Sciences, McMaster University, Hamilton, Ontario, Canada

**Keywords:** Driving, Driving Retirement/Cessation, Fitness to Drive, Occupational Therapy, Transportation

## Abstract

**Background and Objectives:**

In most western countries, older adults depend on private cars for transportation and do not proactively plan for driving cessation. The objective of this review was to examine current research studies outlining effective interventions and strategies to assist older adults during their transition from driver to driving retirement or cessation.

**Research Design and Methods:**

A search was completed across 9 databases using key words and MeSH terms for drivers, cessation of driving, and older adult drivers. Eligibility screening of 9,807 titles and abstracts, followed by a detailed screening of 206 papers, was completed using the Covidence platform. Twelve papers were selected for full-text screen and data extraction, comprising 3 papers with evidence-based intervention programs and 9 papers with evidence-informed strategies.

**Results:**

Three papers met the research criteria of a controlled study for programs that support and facilitate driving cessation for older adults. Nine additional studies were exploratory or descriptive, which outlined strategies that could support older drivers, their families, and/or healthcare professionals during this transition. Driving retirement programs/toolkits are also presented.

**Discussion and Implications:**

The driver retirement programs had promising results, but there were methodological weaknesses within the studies. Strategies extracted contributed to 6 themes: *Reluctance and avoidance* of the topic, *multiple stakeholder involvement* is important, *taking proactive approach* is critical, *refocus the process* away from assessment to proactive planning, *collaborative approach to enable “ownership” of the decision* is needed, and *engage in planning alternative transportation* should be the end result. Meeting the transportation needs of older adults will be essential to support aging in place, out-of-home mobility, and participation, particularly in developed countries where there is such a high dependency on private motor vehicles.

**Translational Significance:** As a means of community mobility, personal vehicles allow older adults the freedom and convenience to engage in social participation. However, there are limited resources for planning when driving in no longer an option, due to advanced age or medical conditions, especially as a result of a crisis. This systematic review describes the three evidence-based driving retirement programs that demonstrated potential for engaging older adults in transportation planning. In addition, evidence-based strategies analyzed under six themes, and seven developed toolkits are described. Stakeholders can access the identified programs, strategies, and outcomes to address this knowledge and program gap for older adults.

The United Nations World Population Prospects (2019) highlights that the global average lifespan continues to increase, with people living longer, and people aged over 65 making up the world’s fastest growing age group. In 2019, global life expectancy was 72.6 years and is projected to increase to 77.1 years by 2050. However, life expectancy in less developed nations continues to lag 7.4 years behind the global average. Longer life expectancy is most profound in those aged 80 years and older, which has grown from 54 million in 1990 to over 143 million in 2019 (United Nations, 2019), with the most aged populations in the developed nations of North America, Europe, and Australia/New Zealand (United Nations, 2019) and Japan having the largest percentage of older adults with 30% (United Nations, 2023).

Longitudinal cohort studies underway in many western countries highlight the high dependency on the personal automobile as the primary and preferred mode of transportation in later life. Two Irish studies found that almost 90% of those aged 65 and older travel most frequently by car (Donoghue et al., 2019) and 72%–76% reported that they drove themselves (Donoghue et al., 2019; Gormley & O’Neill, 2019). Similar patterns have been reported in other countries, including Australia where up to 70% of those aged 65 and older relied on private transport (by motor vehicle) for their out-of-home trips (O’Hern & Oxley, 2015). However, it has been noted that older adults have significantly fewer trips per day than younger people (aged 18–64) with a significant decrease in such trips for those aged 75 years and older (O’Hern & Oxley, 2015). Gormley and O’Neill (2019) found that the number who reported driving themselves began to decline in the 65–69 age category with a notable increase in those aged 75 and older who indicated relying on others with a private car for their transportation needs.

The reliance on access to a private motor vehicle either as a driver or passenger among older adults has also been highlighted in a cross-sectional comparison of older adults across seven countries where 82.1% of those surveyed relied on driving as their primary mode of transport (Unsworth et al., 2022). Interestingly, between 60% and 93% of these respondents reported never using other forms of transportation (e.g., bus, train, tram/streetcar, taxi) regardless of environmental conditions, such as inclement weather (Unsworth et al., 2022). Other studies also indicate that the frequency by which older adults use public transportation is less than 10% for out-of-home trips (Donoghue et al., 2019; O’Hern & Oxley, 2015). In fact, low frequency of using public transportation have been reported across many groups regardless of age or geographic location (i.e., urban or rural; Gormley & O’Neill, 2019).

This combination of increasing global life expectancy, high dependency on the private motor vehicle as the primary mode of transportation, and the low utilization of alternative forms of transportation raises concern as to how older adults will meet their out-of-home transportation needs when they can no longer drive. In an Australian cohort of older adults aged 68–72 years, Anstey et al. (2017) reported a higher proportion of older male drivers than females, and that on average these older adults who were drivers expected to continue driving for another 13 years. The analysis conducted by Foley et al (2002) concluded that older drivers would generally outlive their “driving years” with the estimation that male and female drivers aged 70–74 would be dependent on alternative transportation for 7 years and 10 years, respectively (Foley et al., 2002). However, it is important to understand that driving cessation is not directly related to age per se (Dickerson et al., 2007; Dickerson, Molnar, Bédard, et al., 2019), but the onset of medical conditions (e.g., dementia, Parkinson’s disease, frailty) affecting a person’s physical, sensory, and cognitive abilities combined with possible polypharmacy may necessitate driving cessation (Pomidor, 2019).

Given the high dependency on driving as the primary and preferred mode of transportation among older drivers in many developed countries, it is hardly surprising that driving cessation can have a negative impact on out-of-home engagement. Being able to drive is associated with better mental health and social participation. Older adults who had reduced or stopped driving were at higher risk for depressive symptoms, loneliness, and lower quality of life than those who continued to drive (Donoghue et al., 2019). Studies have shown that those who ceased driving had fewer social networks, were less likely to engage in social leisure activities, and other out-of-home activities than older drivers (Donoghue et al., 2019; Pristavec, 2018).

Interestingly, a qualitative Canadian study highlighted some positive lifestyle changes associated with driving cessation among older adults (Mullen et al., 2017). The participants described how they embraced the challenge of learning to use public transport, possible financial savings accrued with no longer owning and maintaining a car, and feeling relieved, as they found driving challenging and stressful. However, these positive associations were strongly outweighed by the negative associations (Mullen et al., 2017). Overall, the lack of spontaneity and autonomy, constant need for planning ahead, unfamiliarity with public transport, increased dependency on others for transportation, negative impact on interpersonal relationships and social interactions, increased isolation, and loneliness were among the negative consequences of driving cessation that were listed (Mullen et al., 2017). Most noteworthy was that greater negative impact was reported by those who had involuntarily given up driving, whereas those who voluntarily ceased driving tended to cope better with the transition (Mullen et al., 2017). Similar concerns were reported by older adults in western Australia where loss of independence, increased reliance on others, inconvenience, and potential reduction in social and family contact were associated with possible driving cessation (Feng & Meuleners, 2020).

The impact of driving cessation may also be influenced by the person’s ability to independently use alternative transportation due to physical and/or cognitive limitations, but this is also highly dependent on such transport infrastructure being in place (Schofield et al., 2023). Former drivers who were able to use public transit reported more social and leisure activity and stronger social networks than those who relied on others for their transportation (Donoghue et al., 2019). The reliance on others to drive can result in a lack of spontaneity in engaging in out-of-home activities and a tendency for nondrivers had to prioritize what may be considered more essential trips such as medical appointments over what might be considered less important discretionary spontaneous activity for pleasure (Davey, 2007; Mullen et al., 2017).

A lack of planning ahead for driving cessation has been highlighted in a cross-sectional survey of 937 older adults in western Australia (Feng & Meuleners, 2020). Drivers aged 75 and older were more likely to plan for driving cessation than those aged 65–69, as were those drivers who lived alone. An influential trigger in considering driving cessation was when an older driver received a “suggestion” that they may need to stop or limit their driving. While older drivers in these categories may have considered driving cessation, only a quarter who had planned driving cessation had made any actual lifestyle changes that would prepare them to transition to driving retirement (Feng & Meuleners, 2020). Harmon and colleagues (2018) surveyed 874 older adults in the United States and found that three-quarters of them had difficulty believing that they could someday be nondrivers and had not engaged in any planning for their future transportation needs. Only 11% had engaged in any high-level planning for their future transportation needs and only 8% had engaged in any planning for a nondriving future. While a large proportion indicated that engaging in “preplanning” for this transition would be beneficial to help prepare for driving cessation, a higher proportion had not proactively sought out any information or engaged in opportunities to discuss current driving or how to remain mobile if they stopped driving in the future (Harmon et al., 2018).

More recently, Schofield and colleagues (2023) investigated the factors that influence planning for driving retirement. In their review, they found four categories of factors that contain facilitators or barriers that affect retirement planning, which included individual (e.g., sense of burden, who is in control), interpersonal (e.g., family, clinicians), environmental (e.g., location, public transport), and policy (e.g., on-road tests, mandatory reporting). The interaction of these diverse factors, unique for each individual, contributes to the complexity of preparing for driving retirement. While there are some established programs specifically designed to address transition to driving retirement that will be outlined later in this paper, research examining outcomes in this area is limited. Rapoport and colleagues (2017) completed a systematic review investigating intervention studies that facilitated driving cessation in older adults; however, they only reported on three studies perhaps indicative of the lack of robust research in this area. They found some promising effects of the interventions but indicated that caution was warranted due to methodological limitations of their included studies (Rapoport et al., 2017).

With an aging population in many developed countries who are highly dependent on private car for transportation, studies indicate that most older adults are not planning or proactively preparing for a possible transition to nondriving status. There is a significant need to examine the process of transitioning from driving to other forms of transportation that may enable continued levels of community participation and community mobility among older adults. Thus, the overall objective of this review was to examine research that focused on the support provided to older adults during their transition from driving to being a passenger. This review examined existing evidence in three main areas:

(a) Empirical research on outcomes of driving transition programs for older drivers,
(b) Strategies that enable healthcare professionals and others to address driving cessation with older adults, and
(c) Programs for driving cessation that have been cited in the published research.

## Method

### Procedure

Reporting for this review was conducted using the Preferred Reporting Items for Systematic Reviews and Meta-Analysis (PRISMA) 2020 and the PRISMA-S checklists (Page et al., 2021; Rethlefsen et al., 2021). A search strategy including the concept groups: driving, cessation of driving, stroke, or dementia or other terms related to aging and older adults was iteratively developed by members of the project team and a health sciences librarian (A. E. Dickerson, T. Stapleton, J. Bloss). The full search translations can be accessed at: https://thescholarship.ecu.edu/handle/10342/10949. The PubMed search was peer-reviewed by a second health sciences librarian using the PRESS checklist (McGowan et al., 2016). The health sciences librarian ran the search across nine databases inclusive of AMED via EBSCOhost, CINAHL Complete via EBSCOhost, Embase, OT Search, OT Seeker, PsycINFO via EBSCOhost, MEDLINE via PubMed, Scopus, and TRID. No limits or filters were used. A data search was rerun on January 24, 2024, to capture any additional studies from when the systematic review was started.

**Figure 1.**
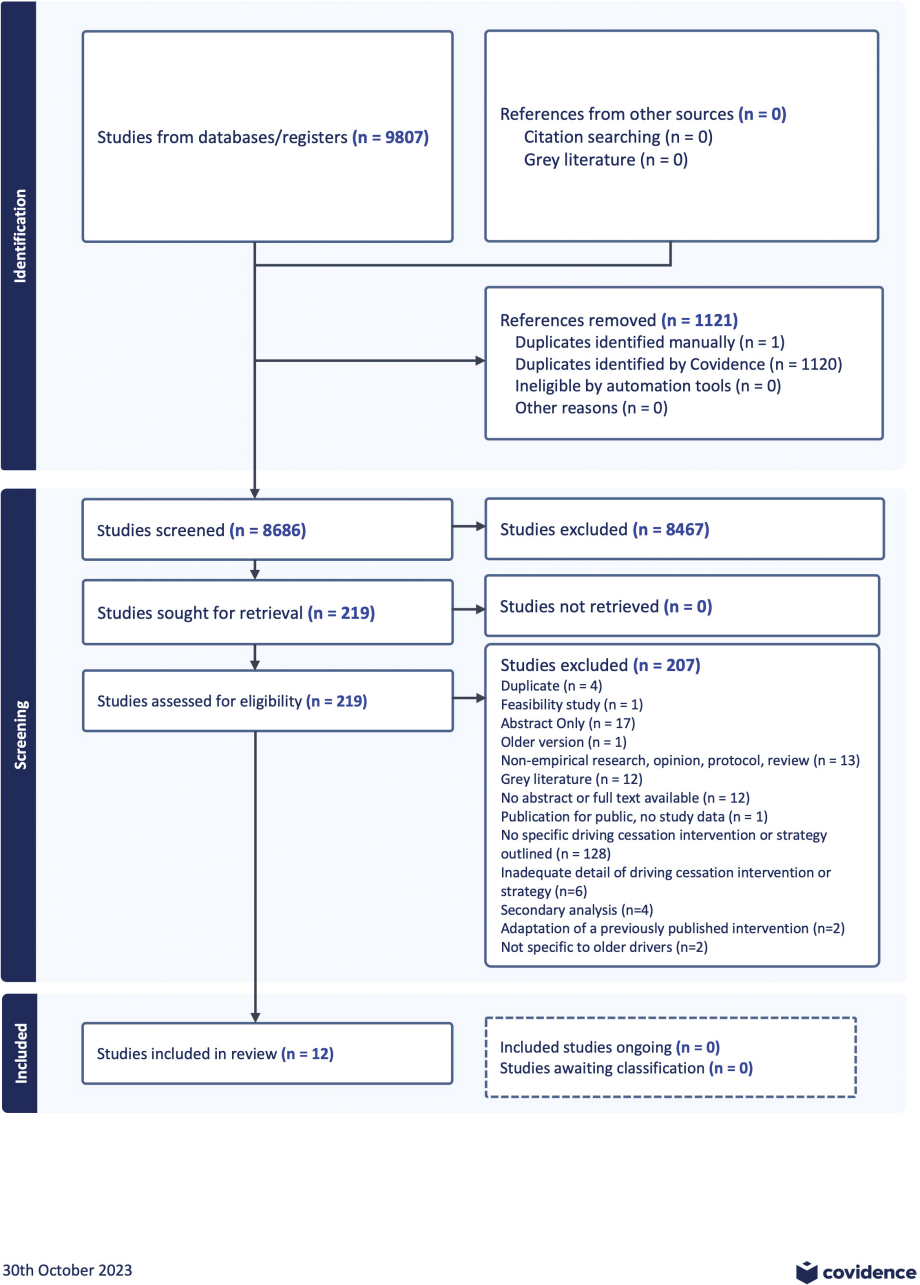
Preferred Reporting Items for Systematic Reviews and Meta-Analysis (PRISMA) of systematic review.

The search records were exported into the Covidence platform and deduplicated. The remaining studies were separately screened by two reviewers for inclusion at the title and abstract level according to the inclusion and exclusion criteria of the study (A. E. Dickerson, T. Stapleton, I. Margot-Cattin, L. Van Niekerk, I. Gelinas, L. Swanepoel, B. Mazer, A.-H. Patomella, M. Choi). Any conflicts during the title and abstract screen were resolved by a third reviewer. Two hundred and six studies were selected for a more detailed title, abstract, and eligibility screen. See Figure 1 for the PRISMA summary of the process.

Eligibility screening was completed by all authors and each study was screened by two reviewers. Any conflicts were resolved by a third reviewer (A. E. Dickerson or T. Stapleton). Only original research articles were included. Studies were excluded during eligibility screen if they contained no specific driving cessation intervention or strategy or if the paper was one of the following: an editorial or opinion piece, only an abstract was available (i.e., a presentation), it was an older version of an included article, gray literature, no study data were included, or a full text could not be found.

Following the eligibility screening, 12 papers were selected for full-text screening and data extraction. Studies that were included for data extraction were classified by the reviewers as either *intervention* or *strategy* papers with all reviewers involved in clarifying what constituted each category. Any conflicts in the classification of a paper were resolved by A. E. Dickerson or T. Stapleton.

*Intervention studies* (*N* = *3*): Research papers that described a direct intervention with the older driver and the outcomes of the program or intervention, which was specifically designed to prepare an older adult for driver retirement; excluding those that only advised the older adult on this transition. Intervention programs were identified as those with a set of coordinated programmatic interventions used in a controlled trial comparing an intervention group and a control group.
*Strategy studies* (*N* = *9*): Research papers that provided guidance for stakeholders (e.g., healthcare professionals, family members, care providers) on how to address driving retirement with older adult drivers were defined as “strategy studies.” Strategies are considered individual methods, approaches, or tactics used to support driving retirement that may be used independently or in concert with other strategies. The recommendations were evidence-based or emerging from research, either quantitative or qualitative, gathered from relevant stakeholders (e.g., older adult drivers, healthcare professionals, physicians, older adult representative organizations).

Two extraction forms were developed: one for interventions and one for strategies. All authors were involved in data extraction, which was performed separately in duplicate using Covidence software, and any conflicts were resolved by consensus (both A. E. Dickerson and T. Stapleton).

## Results

With a systematic search of nine databases, 8,686 records were retrieved after duplications were removed. After screening the abstracts, 219 full-text papers were reviewed with 12 included in this review. We found only three intervention studies that met the inclusion criteria of using a controlled design to examine the outcomes; the same studies identified in a systematic review by Rapoport and colleagues in 2017. In addition to the papers identified as either intervention (*n* = 3) or strategy papers (*n* = 9), a number of papers were identified that outlined structured driving cessation programs. These additional papers provided overviews and information that was neither an intervention study nor strategies identified based on research evidence, but is a copulation of best evidence in terms of *Program Toolkits* (*N* = 7). Program toolkits are strategies put together in one package or website in which the whole or individual parts can be used, but have not been evaluated for effectiveness as whole.

### Intervention Programs

For evidence of what interventions are effective in promoting and facilitating driving cessation for older adults, three papers met the research criteria of a controlled study. Table 1 summarizes these studies (see Supplementary Table 1 for full details). Only three controlled studies were found that met the inclusion criteria—the same three studies as reported by Rapoport et al. (2017) in their systematic review. Specifically, the three studies were conducted in three different countries (e.g., Australia, Canada, United States) with multiple sessions to address preparing for driving cessation with three different populations. All three studies had significant outcomes, but with limitations of small samples and/or high attrition. In Canada, Dobbs et al. (2009) designed and implemented a 16-week driving session support group for individuals with dementia using coping effectiveness training and compared the outcomes to a traditional support group. In the United States, Stern et al. (2008) used psychoeducational and motivational interviewing over four sessions with caregivers of current drivers with cognitive impairment comparing an active intervention group, written materials-only group, and a waitlist by randomized allocation. Many of the materials used were based on the Hartford toolkit materials. From Australia, Liddle et al. (2014) developed an awareness, psychoeducational, and support program for older adults who had to cease driving or planned to cease driving also using a randomized allocation method for the intervention and a waitlist group. None of the studies did well on the Jadad quality report (Rapoport et al., 2017).

**Table 1.** Driving Retirement Programs Evaluated Through Controlled Trials.

Study	Participant criteria	Research approach	Intervention	Outcomes
Dobbs et al., 2009, Canada	Dyads: Individuals with dementia and primary care provider.	Pre–post nonrandomized control group design. 16 weeks of 90-min intervention, five waves over 2 years.	Coping effectiveness training; problem-focused coping for changeable stressors; emotion-focused coping for unchangeable stressors. Control group: Traditional support group for Alzheimer’s disease.	Differences between two groups (*p* < .10)
				GDS: *p* = .03 Quality of life: *p* = .08 Checklist of problems: *p* = .09 Emotional impact: anger: *p* = .09; happy: *p* = .06; surprised: *p* = .10 Likert scale questions: coping with not driving: *p* = .08; recommend group: *p* = .01
Stern et al., 2008, United States	Care partners of individual with cognitive impairment (e.g., spouse, adult child)	Repeated measures with control group. Randomized to one of three groups: (1) active psychoeducational group intervention, (2) written materials only, (3) control. Baseline evaluation: Participant and interviewer blind to assignment.	Active (six sites): Four 2-hr educational sessions using manualized curriculum for each session. Written (five sites): Given materials, resources, and list of driving evaluation programs. Control (three sites): Nothing until after post-test.	Comparison *Self-efficacy*, *p* < .05, active significantly higher than written; written and control the same. *Brief COPE*:
				*Venting*: Active significantly higher than written; written and control the same. *Acceptance*: Active and written are the same and significantly higher than control.
				*Stages of change*: Active significantly higher than written; written and control the same. *Concern about relationship*: Active significantly different (less concern) than written. *Communication*: Active significantly higher than written; *Awareness of agreement*: Active significantly higher than written.
Liddle, et al., 2014, Australia	Older adults over 60 years	Stratified randomized control group (intervention, wait list as control); three time periods	UQDRIVE Program (University of Queensland Driver Retirement Initiative). Awareness component, psychoeducational and support; seven person-centered modules focused on cessation as an intervention.	*Primary*: Post: Intervention group had 5.9 episodes away compared to 4.4 for control group; difference of at least one episode between those with high/low self-rated health and retiring/retired drivers. *Linear regression*: Time 2 significant effect for increased episodes for intervention group, but not maintained at third measurement time. *Secondary*: Intervention group show increased trend in use of transports after intervention, but not maintained. Participants with better health and still driving reported higher satisfaction.

*Notes*: Brief COPE = Coping Orientation to Problems Experienced Inventory; GDS = Geriatric Depression Scale.

### Strategies for Driving Cessation

In this review, we also searched for strategies that support and/or facilitate driving cessation for older adults that appear to be used effectively within medical systems, older adult programs, or stakeholders who support aging adults. Supplementary Table 2 summarizes the nine exploratory or descriptive studies that identified and discussed the complexities of driving cessation and outlined strategies that could assist healthcare professionals and families to address the issue of driving cessation with older drivers. The studies outlined the challenges faced by older drivers, their families, and healthcare professionals in addressing driving cessation and its impact on meeting transportation needs as well as coping with life after driving. Two studies comprised of older adults only; one of these studies used a mail survey consisting of open- and closed-ended questions among older adults (Bryanton & Weeks, 2014; Meuser et al., 2013) and the other study used a mixed approach of an initial focus group followed by a mailed survey (Meuser et al., 2013). The remaining seven studies used either focus groups or individual interviews with a combination of older drivers (current and ex-drivers), family members/informal caregivers, and healthcare professionals. One study had a combination of older adults (drivers and former drivers) and healthcare professionals (Friedland & Rudman, 2009), two studies included older adults, healthcare professionals, and family caregivers (Perkinson et al., 2005; Sinnott et al., 2019), and one study included older adults and family caregivers (Byszewski et al., 2010). The three remaining studies included healthcare professionals and family caregivers (Jouk & Tuokko, 2017; Stasiulis et al., 2020) and one study involved healthcare professionals with input from an older adult representative organization (Byszewski et al., 2013).

All studies that involved older adults included both current and former drivers. Some studies had higher proportions of current drivers; Bryanton and Weeks (2014) sample of 201 older adults comprised of 193 (96%) current drivers, 76% of the 297 survey respondents in Meuser et al.’s (2013) study were current drivers, while 67% of the older adults in Friedland et al.’s (2009) study were current drivers. Byszewski et al. (2010) included 11 older former drivers within their overall sample of 15 (73%). In the studies with mixed sample demographics there was usually a smaller proportion of older drivers and subsequently a smaller proportion of older former drivers (Perkinson et al., 2005; Sinnott et al., 2019). In the studies where a diagnosis was mentioned, all either referred to dementia or cognitive impairment (Byszewski et al., 2010, 2013; Perkinson et al., 2005; Sinnott et al., 2019; Stasiulis et al., 2020; see Supplementary Table 2 for participant demographics).

In a synthesis of these nine studies, anumber of strategies emerged across the nine studies that, if applied in practice, might encourage older adults and healthcare providers to engage more proactively in preparing for the transition from driving to driving cessation and could enhance the process of this difficult decision making for all stakeholders involved. These six themes included using: a cooperative approach involving all relevant stakeholders, a proactive rather than a reactive approach, refocus the process by empowering the retiring driver in the decision-making process of loss of licensure as well as determining their alternative transportation options.

### Driving Retirement Programs

Table 2 summarizes the driving retirement programs developed to assist with driving retirement by experts in the area of older adult and/or transportation research and widely available for use (*N* = 7). Two of the programs (Liddle et al., 2014; Stern et al., 2008) completed at least one research outcome study that is included in the systematic review. Three programs were developed with support from the National Highway Traffic Safety Administration (NHTSA; Dickerson, 2021; National Highway Traffic Safety Administration, 2009; Pomidor, 2019) and two are Canadian online educational and web-based programs that are often referred as “toolkits” (Jouk & Tuokko, 2017; Stasiulis et al., 2023).

**Table 2.** Overview of Currently Available Driving Retirement Preparation Programs/Resource Packs

Name of resource	Overview of content	References in literature
Car Free Me (formerly UQDRIVE)	A seven-module program to address the practical and emotional needs relevant to driving cessation, led by a trained health professional. Is person-centered and covers awareness, adjustment, and practical support. The seven modules specifically include: *Growing older, Driving later in life, Experiences of driving cessation, Adjusting to losses and change, Alternative transport, Lifestyle planning*, and *Advocacy and support*. It was originally designed for all older adults, but has been adapted specifically for those with dementia with changes to Module 1 being *Living with dementia* and Module 2 *Balancing independence and Safety* and an additional module (8) called *Family member/caregiver* to address unlicensed driving, having the conversation, and coping with caregiving. Designed in Australia, it has been in Canada and in the United States. https://carfreeme.com.au/	Liddle et al., 2014; Scott et al., 2019; 2020
At the Crossroads, Hartford Center for Mature Market Excellence, Hartford Insurance	At the Crossroads Intervention was developed as a dementia caregiver group intervention to assist in driving cessation. The workshop is designed for practical application, organized into three 2-hr sessions with nine lessons. It includes an introduction for group leaders, seven appendices of resources, and worksheets for all activities or lessons. The first session is assessing driving ability and activity and includes how the brain changes, warnings signs for drivers with dementia, and how to assess transportation needs. The second session focuses on family cooperation and communication with how to plan conversations collaboratively. The third session focuses on options including making agreements, role of health care providers, and last resort options to stop driving.	Stern et al., 2008
Driving Transitions Education: Tools, Scripts, and Practice Exercises National Highway Traffic Safety Administration and American Society on Aging	This tool was developed as a resource for professionals to have a step-by-step procedure to respond to inquiries about older driver safety, preparing older adults and family members about driving decisions, and discussing transitions from driving to alternatives that maintain community mobility. Section I describes the key messages, how to use the module, and what information a professional needs to have in their area. Section II includes the actual tools and scripts of what to say for 15 topics/modules that range from statistics about older drivers, preparing for the conversation, transportation alternatives, retirement from driving, and dementia and driving. Each module is structured to suggest the presentation time, the audience, objectives, the checklist to have for the presentation, and a written script of what to say. Section III has examples of practice questions and comments for conversations and Section IV has both the resources for the professionals and the public including 1–2 handouts for the presentations. These include caregiver checklist, changes that affect driving, travel skills, and others.	National Highway Traffic Safety Administration, 2009
Clinician’s Guide to Assessing and Counselling Older Drivers, 4th Edition. New York: The American Geriatrics Society; 2019	This Guide is a comprehensive document for primary care providers or other health professionals to assist in assessing and counseling older drivers. Written by an editorial board across medical professions, the Guide has 10 chapters with appendices that offer CTP codes, caregiving information, and resources. The 10 chapters cover an overview of older drivers, screening and assessing for driving risk, intervention strategies, how to use driver rehabilitation, transitioning from driving, ethical and legal issues, state licensing/reporting laws, medical conditions and medications that affect driving, and how to meet the future transportation needs. There are case studies within most chapters and a large array of resources for clinical practice.	Pomidor, 2019
Down the Road Toolkit	This resource was created as an interactive toolkit that aims to promote thought, discussion, and guidance for caregivers about navigating the topic of driving safety and cessation with their family member. It consists of a theater film of three characters, a daughter, son, and grandfather. The Viewer Guide uses the film to address the various issues with discussion questions and background information. The eight themes include: the importance of driving, the assessment process, refreshing skills, risk factors and warning signs, starting the conversation, dealing with resistance, support system, and alternative transportation options. The film, Guidebook and informational cards are downloadable. The informational cards are only useful for Victoria, British Columbia.	Jouk & Tuokko, 2017
Driving and Dementia Roadmap	This online education and web-based toolkit has multicomponents for current and former drivers with dementia and caregivers. It is developed on the Knowledge-to-Action process, and as a “roadmap” has various topics of driving cessation to meet the diverse needs of individuals who need assistance in the decision making and transition to nondriving. https://www.drivinganddementia.ca/	Stasiulis et al., 2023
Plan for the Road Ahead	This online toolkit is dedicated to older adults for the process of transportation planning. It includes two videos to promote transportation planning as part of the retirement process. The website includes links to evidence-based self-assessments and the Assessment of Readiness for Mobility Transition, information about driving evaluations, an online transportation planning tool, financial calculator, and additional resources. https://planfortheroadahead.com	Dickerson, 2021

*Notes*: CTP = Current Procedural Terminology; UQDRIVE = University of Queensland Driver Retirement Initiative.

## Discussion

Our systematic review of the literature found only three intervention studies meeting the criteria for using a controlled design. These were the same three studies identified by Rapoport and colleagues in a 2017 systematic review. Our conclusions are similar, that is, there are some promising effects from these interventions though, unfortunately, all three studies have major methodological limitations including low-quality ratings, also raised in the previous review (see Rapoport et al., 2017). This lack of controlled studies in the intervening 7 years may indicate the difficulty of executing high-quality research and controlled trials in this complex area of practice. Hence, by expanding our review, we included an exhaustive search of identified strategies to address driving cessation as well as current programs for driving retirement preparation available for stakeholder use.

### Strategies to Driving Cessation

#### Reluctance/avoidance

Our findings highlight that there is often a reluctance and avoidance among healthcare professionals, physicians/primary care providers, and family members in broaching the subject of driving cessation (Friedland & Rudman, 2009; Stasiulis et al., 2020). Additionally, in at least one study that we found, some physicians did not see addressing or planning for driving cessation as part of their scope of practice (Friedland & Rudman, 2009). Several papers highlighted the general reluctance among physicians and other healthcare professionals in addressing driving restrictions and driving cessation, which often stemmed from fear of possible adversarial outcomes such as damaging the doctor–patient relationship (Friedland & Rudman, 2009; Sinnott et al., 2019). The potentially devastating consequences on an older adult’s health, quality of life (e.g., depression, isolation), and potentially increasing the burden on family caregivers resulting from the decision that one can no longer driver were raised (Friedland & Rudman, 2009). However, there remains uncertainty and lack of confidence in the underlying processes used to make the decision and final recommendation for driving cessation added to reluctance and unease among physicians (Friedland & Rudman, 2009). Studies also highlighted a general dissatisfaction among older drivers and family/caregivers when there was inadequate input from healthcare professionals (Friedland & Rudman, 2009; Stasiulis et al., 2020). Similar issues were identified by Schofield et al. (2023) in their recent review, the additional risk of litigation (i.e., lack of immunity) may also be a barrier and contribute to the reluctance among some physicians in addressing driving cessation (Silverstein & Barton, 2010). These findings highlight the need for a more proactive and collaborative involvement of healthcare professionals, older drivers, and their caregivers before, during, and after decisions concerning driving cessation.

#### Multiple stakeholder involvement

The burden of managing driver cessation often falls on the older driver’s family. It is primarily family members who have the final responsibility in ensuring the driver adheres to the advice to actually stop driving, provide alternative transportation for the retired driver, and deal with the emotional impact of driving cessation on the driver and themselves (Jouk & Tuokko, 2017; Perkinson et al., 2005). In this review, eight of the nine papers recommended that planning for and managing driving cessation requires the involvement of multiple stakeholders at the time the decision is made that an older driver should no longer be driving as well as in the time period that follows (Bryanton & Weeks, 2014; Byszewski et al., 2010, 2013; Friedland & Rudman, 2009; Jouk & Tuokko, 2017; Perkinson et al., 2005; Sinnott et al., 2019; Stasiulis et al., 2020). Typically, a tripartite stakeholder group comprised of the older driver, family caregivers, and healthcare professionals (e.g., physician, primary care providers) is recommended when addressing and managing the process of driver retirement. The importance of healthcare professionals and family caregivers working in close collaboration was emphasized in order to provide consistent information and advice to the older driver in question (Jouk & Tuokko, 2017) as these frontline stakeholders (e.g., older drivers, family members, healthcare professionals) are typically the ones involved or in proactive planning for driving cessation.

#### Taking proactive approach

Driving cessation decisions often tend to be abrupt, reactive, and a forced decision due to a situation that has escalated to a “crisis” point (Friedland & Rudman, 2009; Stasiulis et al., 2020). There is dissatisfaction with this reactive approach and abrupt notice among all stakeholders, particularly among the older adult drivers who are subjected to the cessation order. Because this reactive approach can be very damaging to the patient–physician relationship, it should be avoided (Byszewski et al., 2010, 2013; Friedland & Rudman, 2009; Sinnott et al., 2019). The general reluctance and avoidance to initiate and engage in proactively addressing driving cessation is a factor contributing to this unsatisfactory reactive approach (Stasiulis et al., 2020).

A proactive approach with early engagement and planning for driving cessation is consistently recommended across the studies. In addition, it was highlighted that healthcare professionals, rather than families, should initiate the conversation about driving and driving cessation (Byszewski et al., 2013; Stasiulis et al., 2020). This resonates with Schofield et al. (2023) review findings that it is the clinician’s responsibility to initially raise the issue. A principal feature of this proactive approach is early engagement of the healthcare professional in open discussion with the older driver about their driving and this engagement should typically include all relevant stakeholders; the older adult driver, concerned family member, and healthcare professional (Byszewski et al., 2010, 2013; Perkinson et al., 2005; Sinnott et al., 2019). Moreover, the conversation initiated by healthcare professionals needs to be well in advance of any concerns about the older drivers’ changing driving ability. This conversation is particularly important in the case of older drivers with a diagnosis of dementia or other cognitive decline. The “advanced planning” approach is recommended, so the discussion of future driving plans occurs before there is an actual problem (Byszewski et al., 2013; Perkinson et al., 2005). Such a conversation should not only commence early, but should be revisited often over the course of all subsequent consultations between the healthcare professional and the older driver (Jouk & Tuokko, 2017; Sinnott et al., 2019).

The provision of education to the older driver and family members regarding the likely progression of a medical condition and how it may affect their ability to drive in the future can also serve as a catalyst or a “warning shot” for driving reduction and eventual cessation (Jouk & Tuokko, 2017; Sinnott et al., 2019). Providing this type of early education and advance planning may enable the older driver and their family to prepare for future driving cessation, thereby enabling a phased transition through gradual reduction of driving, and consideration of possible alternative transportation options in advance of driving cessation (Byszewski et al., 2013; Friedland & Rudman, 2009; Perkinson et al., 2005; Sinnott et al., 2019; Stasiulis et al., 2020). This early proactive approach ensures that current driving and future driving plans are openly discussed and not avoided, and may mitigate some of the emotional turmoil associated with a sudden notice to cease driving.

#### Refocus the process

A shift away from the predominant focus on assessment of fitness to drive is recommended in order to facilitate an approach that encompasses a more proactive engagement in collaborative planning for future driving changes (Byszewski et al., 2013). While healthcare professionals have a responsibility to assess medical fitness to drive, they should also engage in a forward planning approach with older adult drivers to plan for eventual driving cessation and avoid the sudden shock of abrupt cessation notice (Friedland & Rudman, 2009).

It is recommended that this early and ongoing discussion of driving is conducted by the driver’s regular primary care provider or healthcare professional who has ongoing contact with the older driver (Sinnott et al., 2019; Stasiulis et al., 2020). Engaging in a proactive approach to future driving and transportation planning, the healthcare professional can work collaboratively with the older adult driver and focus initially on maintenance of driving while guiding a graded approach of gradual driving restriction in preparation for future driving cessation (Perkinson et al., 2005; Sinnott et al., 2019).

When assessing driving cessation, healthcare professionals such as occupational therapists should use objective tests and measures to inform and support the cessation recommendation (Sinnott et al., 2019). The need to provide clear and unambiguous feedback to the older driver and family is imperative (Byszewski et al., 2010; Friedland & Rudman, 2009; Perkinson et al., 2005). Healthcare professionals should explain how test results relate to driving, why the results are causing concern, and why driving cessation is recommended. In addition to this verbal feedback and explanation, it is also advisable to provide written feedback and information to the older driver (and family) and document what, how, and to whom, information was provided (Byszewski et al., 2013; Perkinson et al., 2005).

To preserve the clinician–patient relationship as much as possible, it is recommended that healthcare professionals emphasize their legal and ethical responsibilities when engaging in driving cessation discussions with the older driver (Byszewski et al., 2010; Friedland & Rudman, 2009; Sinnott et al., 2019). When informing the older driver of the recommendation to cease driving, it may help physicians/healthcare professionals to “depersonalize” the decision. Potentially, the healthcare provider can emphasize that the driving cessation recommendation is based on the interpretation and implementation of the relevant driving regulations or guidelines (Sinnott et al., 2019), and also to emphasize with the patient and family that this process is part of their disease, medical condition, or advanced aging, not an arbitrary decision based on their age.

Knowing the appropriate timing to address driving cessation and the need to assess fitness to drive can be problematic (Byszewski et al., 2013). It is suggested that the time point of transition from very mild to mild, or transition from mild to moderate Alzheimer’s disease are natural junctions to start considering driving and driver assessment, or when the family members begin to raise concerns (Perkinson et al., 2005). The *Assessment of Readiness for Mobility Transition* (ARMT) is a tool designed to measure readiness for mobility transition and may enable individualized planning for mobility transition (Meuser et al., 2013). While the ARMT fails to highlight a specific course of action to be taken to facilitate the transition, it may assist healthcare professionals with identifying if a driver is ready to consider the transition to driving retirement with potential points of discussion and strategies. Subsequently, it may assist with optimal timing to address driving cessation and future transportation planning. Recognizing changes in an older driver’s level of function and progression of their medical condition is another reason why it is important that the primary care providers are aware of changes in their health that can affect on driving (Sinnott et al., 2019).

#### Collaborative approach to enable “ownership” of the decision

Embedding a collaborative partnership approach when addressing transportation needs and planning for future transportation transitions is emphasized across most papers. Healthcare professionals should employ an educational approach focused on encouraging older adult drivers to consider the possible impact of future driving cessation on their life and engage with the older driver in planning ahead and preparing for future transportation (Bryanton & Weeks, 2014; Byszewski et al., 2013; Stasiulis et al., 2020). A collaborative approach involving the healthcare professional, the older adult driver, and the family/caregiver (if appropriate) is recommended with the healthcare professional actively assisting the older adult (and family) to develop self-awareness of changes and signs indicative of the need for driving retirement (Bryanton & Weeks, 2014).

This collaborative process should encompass a shared decision-making approach involving healthcare professionals, the older driver, and their family members. For example, having the driver monitor, appraise, and offer feedback on their own driving ability, collaboratively discussing possible driving restrictions, and having the driver “echo” back the agreed decision assists in fostering some ownership of the decision (Friedland & Rudman, 2009; Sinnott et al., 2019). This type of collaborative partnership approach would potentially enable a voluntary rather than a forced cessation of driving (Byszewski et al., 2013). By engaging in shared decision making, the older adult driver will have more control and ownership of the decision to cease driving rather than being abruptly advised by others to stop (Friedland & Rudman, 2009; Jouk & Tuokko, 2017; Stasiulis et al., 2020). However, this is not without its challenges as some older drivers may be reluctant and resistant to receive feedback on their performance or accept that they may need to make changes to their driving and transportation preferences (Eby et al., 2012).

#### Engage in planning alternative transportation

While some physicians reported that they did not consider engagement in future mobility transition and transportation planning with older adult drivers as part of their scope of practice, older adult drivers do expect input from healthcare professionals in the transition to driving cessation (Friedland & Rudman, 2009). Because there are many negative emotional, psychological, and social consequences associated with driving cessation such as increasing loneliness and dependency on others (Bryanton & Weeks, 2014; Sinnott et al., 2019; Stasiulis et al., 2020), older drivers need to be psychologically and emotionally prepared for driving cessation and its impact on their current lifestyle (Bryanton & Weeks, 2014). Proactively planning for mobility transition and driver retirement should focus on assisting the driver to identify alternative transportation and assist in making the transition to enable continued engagement in meaningful activities, social relationships, and social participation (Bryanton & Weeks, 2014; Sinnott et al., 2019; Stasiulis et al., 2020).

There was consistent recommendation across the studies for proactive involvement of healthcare professionals in alternative transportation and mobility planning with older retiring drivers, and mobility transition planning to mitigate the possible negative consequences following driver retirement (Bryanton & Weeks, 2014; Byszewski et al., 2010; Jouk & Tuokko, 2017; Perkinson et al., 2005; Sinnott et al., 2019; Stasiulis et al., 2020). Older adult drivers considering driving cessation should be provided with information on alternative transportation options. Healthcare professionals, especially occupational therapists, should have local knowledge and are well placed to give advice and direct the driver to other information resources and local community supports (Bryanton & Weeks, 2014; Byszewski et al., 2013; Jouk & Tuokko, 2017; Perkinson et al., 2005).

Healthcare professionals need to also engage with family caregivers in mobility transition planning as family may have to take on the major responsibility for transportation (Byszewski et al., 2010, 2013). Additionally, the impact on the family/caregiver needs to be addressed in this mobility transition (Jouk & Tuokko, 2017; Perkinson et al., 2005). It has been suggested that educational programs to support older drivers preparing for driver retirement should also include some peer element involving others who are retiring or have retired from driving to share experiences and advice to assist retiring drivers to develop a future alternative transportation plan (Bryanton & Weeks, 2014). When planning an individual mobility and transportation plan post-driving cessation, it is suggested that it should be written out in addition to being discussed verbally (Byszewski et al., 2013). The need for healthcare professional to be involved alongside policy makers and community leaders in working collaboratively to advocate and develop alternative transportation options for older adults retiring from driving has been identified (Stasiulis et al., 2020) supporting similar recommendation in the recent review by Schofield et al. (2023). Moreover, as cautioned by Schofield et al. (2023), the alternative transportation options are dependent on transport infrastructure being in place in the environment, which may include diverse levels of assistance and support needed for older adults with and without disabilities as well as vehicle modes and the range of desired destinations (Kerschner & Silverstein, 2018).

### Driving Retirement Programs

Four of the seven current and available programs/toolkits were developed specifically for older adult drivers with dementia. As one of the most common diagnoses of older adults, it is also the most challenging in terms of driving decisions. While drivers with early dementia or mild cognitive impairment may be able to make appropriate driving decisions and judgments necessary to navigate to familiar places, as the disease progresses, the older adult driver loses their higher level cognitive abilities over time (Carr et al., 2019). While operationally still able to “drive” their vehicle, due to loss of judgment and self-awareness (Chen et al., 2020), the older adult driver with moderate to advanced dementia often does not understand why they cannot drive. Thus, it is important to start addressing driving early in the disease process and involving the caregiver. The four programs designed specifically for those experiencing such challenges include: *Car Free Me* (Liddle et al., 2014), *At the Crossroads* (Stern et al., 2008), *Down the Road Toolkit* (Jouk & Tuokko, 2017), and the most recent, the *Driving and Dementia Roadmap* (Stasiulis et al., 2023).

As one of the first programs to be free and made widely available, *At the Crossroads* was promoted through AARP (formerly the American Association of Retired Persons) and other stakeholder associations in the United States, especially after the Stern et al. (2008) study showed positive results for those involved in the intervention over the control (waitlist) group. The intervention was designed as a three-part interactive workshop for dementia caregivers and continues to be widely available with many of its key components (e.g., driving warnings signs, family agreement, transportation plans) currently being used or variation of the forms in circulation. Concurrently, in Australia, the University of Queensland Driver Retirement Initiative (UQDRIVE; Liddle et al., 2007) was developing an occupational therapy-led intervention to prepare older adult drivers in the transition to driving cessation. Liddle et al.’s (2007) program is one of the few programs to have evidence to support efficacy of outcomes. Unlike *At the Crossroads*, UQDRIVE evolved over time with an adaptation to drivers with dementia and a name change to *Car Free Me* (Scott et al., 2019, 2020). Specifically, the session of “growing older” was changed to “living with dementia” and an eighth session was added for the family member/care provider dealing with the driver with dementia. The program also involves a peer support element that has been highlighted as a strategy in this review and others (Schofield et al., 2023). More recently, the *Car Free Me* program has been tailored for and piloted in United States (Peterson et al., 2022) establishing initial support for the program to develop with a peer intervention component. The program has also been translated into French for the French–Canadian context (Pigeon et al., 2020) and used with retiring taxi drivers in Singapore (Chan et al., 2015) and a recent pilot study on adaptation of *Car Free Me* for people with dementia (Scott et al., 2020).

The two other programs addressing dementia and driving were developed in Canada and are primarily web-based. Although both have specific information about reporting, licensing, and transportation options for their jurisdiction in Canada, resources are readily available and appropriate for widespread stakeholder use (Jouk & Tuokko, 2017; Stasiulis et al., 2023). Developed as part of a doctoral dissertation, the *Down the Road* toolkit (Jouk & Tuokko, 2017) is unique as it uses a theater film to illustrate the various issues of driving cessation with characters of including an aging father, an adult daughter concerned about his driving, and the grandson asking questions. An accompanying Guidebook includes background information and discussion questions on eight themes related to driving cessation with informational cards on transportation in Victoria, British Columbia.

In contrast, the other Canadian based toolkit, the *Driving and Dementia Roadmap*, was developed by a team on the behalf of the Canadian Consortium on Neurodegeneration in Aging Driving and Dementia Team (Stasiulis et al., 2023). Using a knowledge-to-action framework, the team developed an educational web-based resource to address both the practical components (e.g., information and awareness, communication, mobility, and community access) and the emotional components (e.g., relationships and role transitions, crisis and conflict, loss and grief, and identity and meaning). The toolkit incorporates other resources already developed (e.g., the Hartford, Alzheimer’s Association) for individuals with dementia, family members or caregivers, and healthcare providers.

As a federal agency mandated to protect all road users in the United States, NHTSA has supported the development of multiple programs and resources to address older drivers. In terms of specific programs or toolkits for driving retirement or cessation, one of the first was the *Driving Transitions Education: Tools, Scripts, and Practice Exercises*, designed in collaboration with the American Society on Aging as a resource for a broad audience of stakeholders dealing with older adult drivers and families (NHTSA, 2009). The toolkit consists of step-by-step procedures for each of the 15 modules. Each module includes key messages, objectives, actual scripts of what to say, checklists, practice questions, and comments for conversation. While somewhat dated, many of the resources are still relevant and certainly paved the way to start discussions about driving decisions. More recently, through a state demonstration project, NHTSA funded a website designed specifically for promoting early transportation planning for older adults (Dickerson, 2021). The website, *Plan for the Road Ahead*, offers similar resources to other resources, but uniquely has the capacity to complete a transportation plan online, complete the ARMT (Meuser et al., 2013), and has two positive, professional 2-min videos to promote transportation planning as similar to retirement planning.

Finally, in 2003, the first edition of *the Physician’s Guide for Assessing and Counselling Older Drivers*, funded by NHTSA, was published in collaboration with the American Medical Association. Now in the fourth edition and changed to the *Clinician’s Guide to Assessing and Counselling Older Drivers* (Pomidor, 2019), the comprehensive document for primary care and other healthcare providers offers extensive resources and remains the leading source of expertise regarding older drivers in the United States.

## Conclusion

The overall aim of this systematic review was to investigate the current research supporting the transition of older adults from self-driving a motor vehicle to driving retirement or cessation. Our review primarily identified research from the United States, Canada, and Australia, which is perhaps not surprising as it may be reflective of the population trends and increasing proportions of older adults in these more developed countries (United Nations, 2019). Because Rapoport et al.’s (2017) systematic review revealed only three papers that demonstrated evidence for outcomes of driving transition programs for older adults, this review was broader by including evidence-based strategies as well as available resources and program toolkits specific to this topic.

The strategies for addressing transportation issues among older drivers highlight the priorities that can be enacted by all healthcare providers. These priorities include encouraging *early* discussion and assisting older adult drivers in planning ahead for driver retirement as a long-term goal for a transitional process rather than a time of medical crisis. Another clear theme that emerged is that healthcare providers of older adults need to change from the predominant focus on assessment of fitness to drive to a more collaborative planning for the transition from driving and assist in how best to manage their out-of-home mobility needs.

As driving and community mobility is well within the occupational therapy scope of practice, occupational therapy practitioners are well positioned to address this issue (World Federation of Occupational Therapists, 2019). Occupational therapists have the skills, knowledge, and clinical judgment to support older adults in this transition process (American Occupational Therapy Association [AOTA], 2020). For some older adult drivers, this may include continued driving with or without restrictions while “trying out” other methods of community mobility such as ride-sharing or e-hailing. Regardless, it is clear the focus should be on health promotion and prevention, that is, highlighting the need to discuss and develop transportation plans early on, especially if the individual is diagnosed with a chronic and/or progressive medical condition, such as dementia. Certainly, many strategies outlined in this review empower healthcare professionals, including occupational therapists to enact such conversations early and focus the conversation on maintaining mobility rather than driving as the only option.

There have been repeated calls within research highlighting the need for driver retirement/cessation programs (Babulal et al., 2019; Dickerson, Molnar, Bedard, et al., 2019; Dickerson, Molnar, Bédard, et al., 2019; Sanford et al., 2020). We have provided an overview of several established programs that focus on driver retirement. Some programs are delivered via in-person group-based intervention while others use online resources to inform and guide older adult drivers, families, care providers, healthcare providers, and other stakeholders (e.g., law enforcement, state licensing agencies, senior service providers). However, there has been limited research on the outcomes of these programs evidenced by the lack of additional research because the previous similar review was undertaken over 5 years ago (i.e., Rapport et al., 2017). It may be that a randomized controlled trial (RCT) is not appropriate for this type of program due to the complexity of the issue. However, there are many complex issues that can be examined using RCTs and other methods, such as dementia prevention, dementia treatment, and assessment of fitness to drive. The more likely explanation for the lack of evidence is that large-scale funding would be necessary to undertake such an evaluation as well as a major paradigm shift from motor vehicles being the primary mode of transportation to the use of public transportation and/or share resources for driving. A successful program requires the solution of how retired drivers will maintain community mobility for their quality of life and social participation. Obtaining such funding would require collaboration across public and private sectors, which is challenging to coordinate due to current fiscal constraints.

In a recent synthesis of driving cessation and dementia, Sanford and colleagues (2020) outlined many of the same strategies of this review such as good communication, advanced planning, and emotional support when stopping driving. Moreover, recent studies have explored facilitators and barriers influencing driving retirement from the perspective of healthcare professionals (Liddle et al., 2023) and through the lens of a health promotion model (Schofield et al., 2023), both highlighting the complexity of the process that varies with each individual. This finding is not a surprise as the same themes and strategies have emerged in all programming developed to support driving retirement/cessation programs. Given their skill set and focus, occupational therapists are positioned to lead such efforts within driving evaluations and rehabilitation (Pomidor, 2019). While there are some examples of occupational therapy interventions in older driver education and retirement (Dickerson & Schold Davis, 2020), more effort is needed if the profession wants to highlight its distinct value in providing critical support to maintain health and well-being, quality of life, functional mobility, and social participation.

In summary, given the global population trends, meeting the transportation needs of older adults will be essential in enabling aging in place and supporting the out-of-home mobility and participation of older people, particularly in the developed countries when there is such a high dependency on private motor vehicles. The biopsychosocial model of functioning and disability of the International Classification of Functioning, Disability and Health (ICF) has been used to measure barriers of transportation by older adults (Schuler & D’Souza, 2021). The ICF highlights the relevance of the inclusion of transportation within healthcare provision to enable participation in community, social, and civic life. While the focus of this current review was on older adult drivers and how to prepare for future transportation needs, these findings are also applicable to people with congenital or acquired disabilities across the lifespan who need assistance with transportation planning as they age and/or acquire a condition that precludes driving. Transportation planning is clearly needed for all persons, especially in a period of time with global efforts to decrease fossil fuels, evolving technological changes, and changing ways of delivering products or services while trying hard to maintain social connections and engagement.

## Supplementary Material

igae054_suppl_Supplementary_Material

## Data Availability

This study was not preregistered. Our “data” are a systematic review that has all the “data” in the tables for the references used in the review. There is a link in the paper to the “terms” used in the search.
