# Supplementary material for: A Systematic Review of Effective Interventions and Strategies to Support the Transition of Older Adults From Driving to Driving Retirement/Cessation
Source: Innov Aging. 2024 Jun 3;8(6):igae054. doi: 10.1093/geroni/igae054 (PMC11212369; doi:10.1093/geroni/igae054)
Supplement: igae054_suppl_Supplementary_Material [file igae054_suppl_supplementary_material.docx]

***Innovation in Aging* Supplementary Material: Dickerson et al. A systematic review of effective interventions and strategies to support the transition of older adults from driving to driving retirement/cessation.**

**Supplementary Table 1. Driving retirement programs evaluated through controlled trials (full details).**

| **Study** | **Participant Criteria** | **Demographics** | **Research Approach** | **Intervention** | **Outcomes** | **Fidelity & Utility** |
| --- | --- | --- | --- | --- | --- | --- |
| Dobbs et al., 2009 (Canada) | Dyads: Individuals with dementia and primary care provider  *Inclusion:*  -Diagnosis of dementia, early stage  -Able to participate in group support  -Community-living  -Loss of driving privileges & difficulty with the loss  -English speaking  -Not in other therapy | ***Intervention***   - n=28 - Age: 77+7.1 years - 57% male - 79% high school education - Driving: 54+12 years   ***Control group:***   - n=19 - No significant differences | Pre-post nonrandomized control group design.  16 weeks of 90-minute intervention, 5 waves over 2 years  ***Outcome measures:***   - Geriatric Depression Scale (GDS) - Quality of Life (QOL-AD) - Revised Memory & Behavior Problems Checklist - Pleasant Events Schedule - Visual Analogue Scale for emotional effects of license revocation | Coping Effectiveness Training; Problem-focused coping for changeable stressors; Emotion-focused coping for unchangeable stressors  ***Control group:*** Traditional support group for Alzheimer’s Disease | Differences between two groups (p<.10)   - GDS: p=0.03 - Quality of life: p=0.08 - Checklist of problems: p=0.09 - Emotional impact: Anger: p= 0.09; Happy: p=0.06; Surprised: p=0.10 - Likert scale questions: Coping with not driving: p=0.08; Recommend group: p= 0.01 | Driving cessation support groups may be effective in eliminating negative consequences of driving cessation.  **Limitations:** Study used a p = 0.10.  If p=0.05, there would be few significant differences: Some outcome measures not established.  ***Utility***  Not explicitly described; Resources for using program only available from authors, makes it difficult to replicate. |
| Stern, et al., 2008 (USA) | Care partners of individual with cognitive impairment (e.g., spouse, adult child)  *Inclusion:*  -Driven two trips per week  -Played role in driving decision  -At least 4 hours direct contact with care recipient  -English speaking  -Willing & available | Total N= 66  ***Active Intervention***   - n=31 - Age: 62.8+10.9 years - 90% female - Relationship:   Spouse 74%, adult child 23%, other 3%  ***Written materials***   - n=23 - Age=68.4+12.4 years - 96% female - Relationship:   Spouse 70%, adult child 17%, other 13%  ***Control***   - n=12 - Age: 61.8+13.7 years - 82% female - Relationship:   Spouse 55%, adult child 46%, other 0% | Repeated measures with control group  Randomized to one of three groups:  1) Active psycho-educational group intervention  2) written materials only  3) control  Baseline evaluation: Participant & interviewer blind to assignment.  ***Outcome measures:***   - Self-efficacy Questionnaire, 7 items (10 point scale) - Brief COPE, coping scale, 28 item with 14 subscales - Stages of change, 2 items, 5 point scale - Concern about relationship, 2 Y/N questions - Communication with about driving - Y/N - Awareness of agreement- Y/N | Active=6 sites  Written= 5 sites  Control=3 sites  ***Active:*** Four 2hr educational sessions using manualized curriculum for each session  ***Written****:* Given materials, resources, & list of driving evaluation programs  ***Control***: Nothing until after posttest | ***Comparison***  *Self Efficacy,* p<.05, Active significantly higher than Written; Written & Control the same.  *Brief COPE:*  *Venting:* Active significantly higher than Written; Written & Control the same. *Acceptance:* Active & Written are the same and significantly higher than Control  *Stages of Change:*  Active significantly higher than Written; Written & Control the same.  *Concern about relationship:* Active significantly different (less concern) than Written;  *Communication:* Active significantly higher than Written; *Awareness of agreement:* Active significantly higher than Written | Videotaped sessions studied to determine consistency across groups.  One group leader: consistency  **Limitations:**  Small samples, especially control; Had to agree to participate regardless the group assignment; Sample likely not generalizable.  ***Utility:***  Program outlined in paper with manualized program developed and available.  Not necessarily designed to assist in cessation of driving but demonstrated efficacy in doing so from perspective of care providers. Program available and accessible for replication. |
| Liddle, et al., 2014 (Australia) | Older adults over 60 years.  *Inclusion:*  -Current driving cessation needs  -Live within program area and available  -Provide consent  *Exclusion:*  -Plans to return to driving  -Cognitive, physical, or communication impairment | ***Intervention:*** N=67  ***Control:*** n=64  Matched on age, gender, health status  Age: 78.9+7.6 years  Gender: 26% male  Retired drivers 52%  No significant differences in gender, driving status, self-rated health for dropouts compared to those who completed, however, significant difference in age (dropouts significantly older and more likely from control group). | Stratified randomized control group;  (Intervention, wait list as control); 3 time periods    ***Outcome Measures:***  ***Primary***   - Episodes of leaving the home per week, survey, & interview   ***Secondary:***   - Modes of transportation, satisfaction (5-point scale) - Community mobility self-efficacy (10-point scale). | UQDRIVE Program (University of Queensland Driver Retirement Initiative)  Awareness component, psychoeducational & support; 7 person centered modules focused on cessation as an intervention. | *Primary:*  Post: Intervention group had 5.9 episodes away compared to 4.4 for control group;  Difference of at least 1 episode between those with high/low self-rated health & retiring/retired drivers.  *Linear regression:* Time 2 significant effect for increased episodes for intervention group, but not maintained at 3^rd^ measurement time.  *Secondary:* Intervention group show increased trend in use of transports after intervention, but not maintained.  Participants with better health & still driving reported higher satisfaction. | *Fidelity:* Blinded assessor collected outcome measures.  **Limitations:**  Control group was referred if needed & may have received services; Recruitment also included drivers who planned to stop; High attrition; Outcome measures difficult & complex to measure.  ***Utility:***  Program only briefly described, but others paper do describe it more explicitly.  Facilitated by trained health professional & peer leader using standardized manual. |

**Supplementary Table 2. Studies which identify and/or describe strategies to address driver retirement/cessation.**

| **Study** | **Focus** | **Participants** | **Approach** | **Description of the Strategy** |
| --- | --- | --- | --- | --- |
| Bryanton & Weeks, 2014  (Canada) | Opinions, suggestions about what is needed in a driver retirement program from older adult perspective. | N=201 adults   - 108 male, 93 female - Age: range 71-96 years - 193 (96%) were current drivers | Mail Survey consisting of both open and closed ended questions | Recommendation of topics to be included in a driver retirement program:   - Education to understand the impact that driving retirement may have on older adults’ lives and how to prepare psychologically for changes. - Coping after driving retirement from peers who had transitioned to driver retirement. - Provision of information for alternative forms of transportation (e.g., low-cost/no-cost transportation options). - Education on how to access and use alternative transportation and accepting transportation assistance. - Finance comparison of costs associated with car ownership versus costs of accessing alternative transportation. - Information for retiring drivers living in rural areas. - Self-awareness to recognize when driving retirement is needed. |
| Byszewski et al., 2013 (Canada) | Developed a toolkit designed for persons in early stage of dementia and caregivers for transition to driving cessation. | Information was gathered from existing resources, reviewed by members of Alzheimer’s society chapters. | Addressed the scope of the problem, aims of the toolkit, toolkit sections: General information, assessment procedures and multiple resources | - Advance planning: Begin discussions about driving early to avoid sudden recommendation to stop. - Proactive approach, plan ahead for voluntary cessation of driving, timing of decision, access to transportation and mobility options, and consult with all involved. - Focus on preparation for driving retirement, rather than assessment using collaborative approach, problem solve to maintain quality of life. - Use family and community supports to develop alternative transportation and mobility plan. - Toolkit includes multiple resources. |
| Byszewski et al., 2010 (Canada) | Perceptions of persons with dementia and caregivers on experience with recommendation of driving cessation. | N=15 persons with dementia   - 8 female, 7 male - Age: mean 81 years - Caregivers: 5 spouses, 8 adult child, 2 other relatives - 11 not driving - 4 active drivers | Qualitative descriptive study | - Start conversations early to mitigate emotional turmoil associated with sudden cessation. - Provide education and feedback, in both verbal and written form, on how test results impact driving performance and how it informs the decision. - Prepare for the emotional reaction of patients and caregivers to the recommendation of driving cessation. Balance delivering the decision by being supportive but explain legal and ethical responsibility. - Be knowledgeable and prepared to provide alternative transportation options to assist with transition. - Involve family/caregiver in the discussion. |
| Friedland & Rudman 2009 (Canada) | Perceptions of barriers and facilitators to dialogue about driving and driving cessation. | N=99   - 29 late middle age; age: range 55-64, mean 59.6 years - 24 older adult drivers; age: range 66-92, mean 75.5 years - 26 older adult ex-drivers,age: range 65-94, mean age 81.8 years - 20 physicians | Secondary analysis of focus groups  Older adults grouped by age and driving status  Physicians separate | **Barriers:**   - Reluctance among physicians, family members and older adults to broach the topic of driving due to fear of: Adversarial outcome, loss of independence, negative impact on existing relationship, negative impact on health, creating dependency and increasing burden on family and friends. - Reluctance to plan creates burden and sudden cessation. - Physicians’ uncertainty and lack of confidence in understanding concerning fitness to drive; they don’t feel it is their role.   **Facilitators:**   - Proactive approach needed. - Feedback in a collaborative manner involving older adult for ownership and control of the decision. - Provide feedback in a non-confrontational manner, physician to emphasize their legal responsibility. - Older adults expect family members and healthcare professionals involvement. - Address driving cessation in a gradual manner over time, not abrupt notice. - Early interventions aimed with anticipation of driving restrictions/cessation facilitate coping and adaptation. |
| Jouk & Toukko, 2017 (Canada) | Development of toolkit to support dementia caregivers about driving. | N=17 caregivers  **Informal:**   - n=8 - 88% female - age: range 59-84 years - Informal caregiver for 3-6 years   **Formal:**   - n=9 - nurses (44%), occupational therapists (33%), registered care aids (22%) | Focus groups and individual telephone interviews | Highlights major themes/content areas : (1) the importance of driving, (2) the assessment process, (3) information about refresher courses and driving schools, (4) risk factors and warning signs, (5) starting the conversation, (6) dealing with resistance, (7) the need for support, and (8) alternative transportation options.  Caregivers wanted to know and recognize the risk factors of unsafe driving.   - Emphasis on family/caregivers and health professionals working together to provide consistent information to driver. - Begin conversation early and revisit often. - Involve the driver with dementia in the conversation to provide a sense of autonomy in the decision-making. - Family and healthcare professionals work together to manage possible resistance, anger or aggression. - Caregiver supports needed when dealing with driving cessation, both practical and emotional. - Information on alternative transportation options provided. |
| Meuser et al., 2013 (USA) | Validation of the Assessment of Readiness for Mobility Transitions (ARMT). | N=297 older adults   - 78% female - Age: mean 71 years; range 55-95 years - 76% drivers | 87 readiness items compared to other standardized validity measures to be refined to 24 items for tool. | - Four readiness types: Anticipatory anxiety, Perceived burden, Avoidance, and Adverse situations. - Higher scores indicate less *readiness* to manage a mobility transition. - Scores can measure change and the three groups (high readiness, mixed readiness, and low readiness) offer a description of each level and questions for consideration and discussion. - ARMT may be useful for healthcare professionals to begin the conversation about transportation planning and help identify when a person is amenable/ready to discuss planning for driver retirement; Different approach needs to be taken with high scorers (less ready) and low scorers (more ready). |
| Perkinson et al., 2005 (USA) | Identify and compare beliefs/perceptions concerning dementia and driving, effective strategies to limit/cease driving. | N=68 participants:   - 10 older adult advocates - 8 non-physicians healthcare - 8 transportation /law enforcement - 6 physicians with expertise in dementia - 8 primary care providers - 9 current drivers - 5 prior drivers - 14 caregivers | 10 two hour focus groups, separate focus groups for each grouping | Recognition that family members of person with dementia have responsibility with limiting/stopping driving.  Specialist’ support is essential to assist in the process.  Persons with mild/very mild dementia may be able to continue driving, but at the transition from mild to moderate AD is the crucial time for intervention by healthcare professionals.  **Strategies:**   - Healthcare professionals must assist family members in dealing with driving and dementia. - Invoke outside authority figures as appropriate and if required. - Provide feedback to the driver on their driving skills. - Start driving conversations in the early stages to prepare driver and family to begin driving restriction and eventual cessation. - Gather concrete evidence to highlight need to stop driving, get formal driving evaluation. - If driving cessation is indicated by physician, must give clear written instructions about not driving. - Remove opportunity to drive, such as remove car, disable the car, or change keys. - Appreciate the significance of driving and make efforts to preserve the driver’s dignity. - Find a substitute and advise on alternative transportation. |
| Sinnott et al., 2019 (Ireland) | Explore general practitioners (GP) and persons with cognitive impairments’ experiences about fitness to drive evaluations. | N=18   - 12 general practitioners - 1 care provider - 5 persons with cognitive impairments   Driving status of participants with dementia (n=5):   - 1 awaiting evaluation - 2 not fit to drive - 2 fit to drive | Semi-structured qualitative interviews | - With cognitive impairment, practitioner should proactively introduce fitness to drive; while currently fit to drive, person needs to prepare for future changes in driving status. - Should be addressed by the practitioner who has relationship with the client/patient. - Driving should be routine discussion for all persons with cognitive impairments. - Begin fitness to drive conversation early and revisit fitness to drive over multiple visits. - Unplanned fitness to drive decisions can be contentious and damaging to patient-physician relationship. - Patient collaboration in preparation for driving cessation with focus on maintenance of driving as long as possible. - Use reflection with driving discussion, ask about self-perceptions/self-appraisal of driving ability, use self-imposed restrictions to trigger self-awareness and insight and offer ownership of the decision back to driver. - Depersonalize the not fit to drive decision using guidelines/ legislation; blame the guidelines to preserve the patient-physician relationship. - Use objective cognitive tests/measures in the assessment and/or involve others in the decision-making process. - Acknowledge the emotional impact of driving cessation on quality of life. - Patients reported not feeling supported or sufficiently assisted to manage negative consequences of driving cessation. |
| Stasiulis et al., 2020 (Canada) | Examine the context-specific factors relevant to the effective implementation of a driving cessation toolkit. | N=15   - 14 female - Alzheimer Society staff members across 4 Canadian provinces - Range of experience: 1-14 years | Qualitative using semi structured interviews  Establishing how organizational resources or supports address the challenges that drivers and former drivers with dementia and carers experience | - Driving cessation among people with dementia is recognised as a very important issue yet it continues to be largely avoided at individual and system level. - A lack of awareness and knowledge about how dementia impacts a person’s driving ability among clients and their families led to delays in considering possible driving cessation. Thus, need to provide this information with resources at different stages. - Recognition that clients with dementia and their family carers often avoid discussing driving until a crisis when immediate cessation is recommended. Service providers is reactive instead of proactive. Therefore, importance of early conversation about driving cessation with person with dementia and physician is needed to help prepare and plan for this transition. - Provide education, support and strategies in supportive approach to enhance their strengths and capabilities to help the person with dementia and family members to adjust and manage the practical and emotional challenges of driving cessation. - Provision of strategies for family members and health care providers to initiate the conversation. - Education and training of health care providers with standardized guidelines outlining the roles and responsibilities in addressing and managing driving cessation. - Resources aimed at supporting the emotional impact of cessation, address the social needs, maintenance of involvement in meaningful activities and social relationships through alternative transportation strategies. - Need for policymakers, clinicians and community leaders to work together to develop affordable alternative transportation options to driving. |
